# Evidence for Post-Translational Processing of Vascular Endothelial (VE)-Cadherin in Brain Tumors: Towards a Candidate Biomarker

**DOI:** 10.1371/journal.pone.0080056

**Published:** 2013-12-16

**Authors:** Isabelle Vilgrain, Adama Sidibé, Helena Polena, Francine Cand, Tiphaine Mannic, Mélanie Arboleas, Sandra Boccard, Antoine Baudet, Danielle Gulino-Debrac, Laurence Bouillet, Jean-Louis Quesada, Christophe Mendoza, Jean-François Lebas, Laurent Pelletier, François Berger

**Affiliations:** 1 INSERM, Unit 1036, Biology of Cancer and Infection, Grenoble, France; 2 UJF-Grenoble 1, Biology of Cancer and Infection, Grenoble, France; 3 CEA, DSV/iRTSV, Biology of Cancer and Infection, Grenoble, France; 4 Grenoble University Hospital, Division of Internal Medicine, Grenoble, France; 5 INSERM 003, Clinical Investigation Center, Grenoble University Hospital, Grenoble, France; 6 Grenoble University Hospital, Division of Radiology, Grenoble, France; 7 INSERM, Unit 836 Brain Nanomedicine, Grenoble Neurosciences Institut Grenoble, Grenoble, France; 8 Joseph Fourier University, Medicine School, Saint-Martin-d'Hères, France; 9 Grenoble University Hospital, Biology and Pathology Institute, Grenoble, France; 10 Grenoble University Hospital, Division of Oncology, Grenoble, France; Indiana University College of Medicine, United States of America

## Abstract

Vessel abnormalities are among the most important features in malignant glioma. Vascular endothelial (VE)-cadherin is of major importance for vascular integrity. Upon cytokine challenge, VE-cadherin structural modifications have been described including tyrosine phosphorylation and cleavage. The goal of this study was to examine whether these events occurred in human glioma vessels. We demonstrated that VE-cadherin is highly expressed in human glioma tissue and tyrosine phosphorylated at site Y^685^, a site previously found phosphorylated upon VEGF challenge, via Src activation. *In vitro* experiments showed that VEGF-induced VE-cadherin phosphorylation, preceded the cleavage of its extracellular adhesive domain (sVE, 90 kDa). Interestingly, metalloproteases (MMPs) secreted by glioma cell lines were responsible for sVE release. Because VEGF and MMPs are important components of tumor microenvironment, we hypothesized that VE-cadherin proteolysis might occur in human brain tumors. Analysis of glioma patient sera prior treatment confirmed the presence of sVE in bloodstream. Furthermore, sVE levels studied in a cohort of 53 glioma patients were significantly predictive of the overall survival at three years (HR 0.13 [0.04; 0.40] p≤0.001), irrespective to histopathological grade of tumors. Altogether, these results suggest that VE-cadherin structural modifications should be examined as candidate biomarkers of tumor vessel abnormalities, with promising applications in oncology.

## Introduction

Primary brain tumors are one of the most aggressive forms of human cancer [1]. While combination of radiotherapy and Temodar chemotherapy significantly improved survival [2], glioblastomas are still associated with a very poor prognosis. 

Neovascularization is one of the most important morphologic features in malignant glioma. It is part of the histologic diagnostic criteria in the current WHO classification scheme and is associated with poor prognosis [3]. Tumor vasculature [4] is highly aberrant, incomplete, and tortuous, thereby creating some areas of hypoxia, acidosis, and peritumor edema [5]. Several studies have shown that increased vascular permeability was correlated with higher grades of tumors and with elevated mitotic index of tumor cells [6]. However, it seems that the contrast enhancement observed in tumors by magnetic resonance imaging (MRI) should not be considered as the unique factor reflecting the tumor malignancy. Indeed, the high grade gliomas that account for 30% of all gliomas have no contrast enhancement in MRI, whereas 16% of low grade gliomas also present the contrast enhancement [7]. Thus it is of major importance to improve the characterization of capillary network in these tumors. Vascular endothelial (VE)-cadherin is an endothelial specific cadherin localized at adherens intercellular junctions of vascular endothelial cells [8]. Unlike most endothelial markers, VE-cadherin is not found in blood cells nor in hematopoietic precursors. VE-cadherin has been shown to play important roles in the establishment and maintenance of endothelium integrity. The importance of the extracellular domain of VE-cadherin in the control of permeability was shown in mice injected with antibodies directed against this domain. Within 24 hours, the mice died due disassembly of the vasculature, and hemorrhage [9]. The cytoplasmic domain of VE-cadherin is also involved in increased permeability when subjected to tyrosine (Y) phosphorylation. Indeed, Vascular Endothelial Growth Factor (VEGF)[10], as well as inflammatory mediators [11,12], induced VE-cadherin tyrosine phosphorylation and endothelial cell-cell dissociation. The first observation of VE-cadherin tyrosine phosphorylation *in vivo* was reported in two endocrine glands expressing VEGF upon hormonal control in the ovary and uterus, [13,14]. In the same study, VE-cadherin was found to be associated *in vivo* with the tyrosine kinase Src and VEGFR-2 in these organs [13]. *In vitro*, our group demonstrated that Src kinase was responsible for VE-cadherin tyrosine phosphorylation at site tyrosine 685 (Y^685^) in HUVECs upon VEGF challenge; a process associated with VEGF-induced endothelial cell migration [15]. Recently, we demonstrated Tumor Necrosis Factor alpha (TNF-α), and Bradykinin, respectively involved in rheumatoid arthritis and hereditary angioedema induced the cleavage of VE-cadherin ecto-domain (named soluble VE-cadherin or sVE, 90 kDa) in a Src dependent manner, [16,17]. Of importance, sVE was detected in patient serum and found to be a marker of HAE attack and RA disease activity [16,17]. This data indicate that VE-cadherin modifications (ie: phosphorylation and cleavage) are of major interest in vascular permeability, angiogenesis and inflammation.

 In tumor angiogenesis, these structural modifications of VE-cadherin have never been explored. However, it is known that blood vessels in tumors are unusually shaped and present cellular abnormalities, in particular at cell-cell junctions [18]. Because discovering new biomarkers is a main challenge in malignant glioma, and given the well-known role of VE-cadherin in stabilizing endothelial cell junctions, the goal of the present study was to characterize the post-translational processing of the protein in human brain tumors.

## Materials and Methods

### Reagents

Leupeptin, pepstatin A, Triton X-100 were purchased from Sigma-Aldrich (Saint Louis, Missouri) and sodium orthovanadate, H_2_O_2_, the MMP inhibitor GM6001 from Sigma-Aldrich, and Src inhibitor PP2 from Calbiochem. Enhanced chemiluminescence (ECL) detection reagents were purchased from Perkin-Elmer (Courtaboeuf, France), nitrocellulose from Schleicher and Schuell (Ecquevilly, France). 

### Antibodies

The polyclonal anti-humanVE-cadherin cytoplamic domain (C19) was from Santa Cruz Biotechnology (Santa Cruz, USA), the monoclonal anti-human VE-cadherin extracellular fragment (clone BV9) from Abcam, the monoclonal anti-phosphotyrosine 4G10 from Millipore, the polyclonal anti-Cad3 antibody against Cad3 domain of human VE-cadherin [19]. The anti-p^658^ and anti-p^731^ VE-cadherin antibodies were from Biosources (CA-USA), and antibodies to Src, p^418^Src, Csk were from Invitrogen (USA). Horseradish peroxidase-conjugated secondary antibodies were from Bio-Rad Laboratories.

### Ethics

All patients enrolled in this trial provided written informed consent. Brain tumor patients enrolled in the Protocol#010960 at Grenoble University Hospital between March 2001 and December 2004 had blood sampling at their first radiological investigation, in accordance with the French legislation. Tumor samples were collected during curative resectional surgery in the Neurosurgery division of Grenoble hospital and frozen (-80°C) after surgery. Tissue and blood samples were stored for scientific research in a biological resources repository (Centre de Ressources Biologiques, Grenoble Hospital), according to national ethical guidelines. Tissue banking and research conduct was approved by the Ministry of Research (approval AC-2010-1129).

### Patients for tissue analysis

The pathological classification of tumor tissues and the stage of cancer were determined according to the WHO classification [3]. Non-tumor human brain tissues (N) were surgically obtained from a patient presenting epilepsy of the right frontal region that had been evolving for several years. Analyzed tissues corresponded to grade IV (n=7), grade III (n=4) and cortectomy (n=1). 

### Tumor tissue extraction

All procedures were performed at 0-4°C. The frozen tissues ( 200 mg) were homogenized in ice-cold lysis buffer (1:20 w/vol) containing 0.5% Triton X-100, using a Potter-Elvehjem apparatus with a Teflon pestle. The homogenate was centrifuged at 15,000 rpm for 45 min. Protein determination was performed using the bicinchoninic acid protein assay kit (Fischer-Scientific, France) with bovine serum albumin as the standard. 

### Immunofluorescence

Tissue sections (10 μm thick) were processed as previously described [13]. 

### Cell culture

Human Umbilical Vein ECs (HUVECs) were grown to confluence in M199 medium supplemented with 10% fetal calf serum (FCS) and 2% low serum growth supplement (Cascade Biologics) as previously described ([20]) They were transfected with siRNAs as previously described [17]. LN229 and U87 were obtained from the ATCC. The cells were grown in 10% SVF Dubelcco’s Modified Eagle medium 4.5 g/L glucose. The medium were collected after serum starvation for the analysis of their content in metalloproteinases activities.

### Extraction, immunoprecipitation and western blotting

Protein extraction and analysis were performed as previously described [17].

### Soluble VE-cadherin analysis in conditioned media

The HUVECs conditioned media were concentrated approximately ten-fold using Centriprep Centrifugal Filter Units with an Ultracel YM-50membrane (Millipore). Western blotting was performed for protein analysis in each sample.The volume of conditioned media analyzed were adjusted according to the corresponding cell number. The relative amounts of extracellular VE-cadherin were measured by densitometry of autoradiographs using the NIH Image J software program.

### Detection of MMPs using gelatin zymography

After three washes, the LN229 and U87 cells were cultured without serum and collected after 24 h. After centrifugation at 15,000 rpm, 3 mL of conditioned media was concentrated to 50 μL using centriprep tubes. An aliquot (5 μL) of the concentrated culture medium was subjected to SDS-PAGE in a gel containing 1 mg/mL gelatin. The gels were then incubated in 2.5% Triton X-100 and rinsed in distilled H_2_O. Gels were further incubated at 37 °C for 20 hours in 20 mM NaCl, 5 mM CaCl_2_, 0.02% Brij-35, 50 mM Tris-HCl buffer, pH 7.6, then stained with 0.1% Coomassie Brilliant blue R-250 and destained in 10% acetic acid, 30% methanol in H_2_O.

### Deglycosylation Assay

20 μl of serum diluted 1 to 40 in PBS-containing 0.5% Triton X-100 and 20 μg/ml leupeptin were resuspended in 80 μl of PGNase buffer. After addition of 5 μl of denaturation solution (2% SDS and 1M 2ß-Mercaptoethanol), the mixture was incubated at 100°C for 5 min followed by addition of 5 μl of detergent solution (15% solution of IGEPAL CA 630). 35 μl of this mixture were then incubated overnight at 37°C with 0.15 units/ml PGNase F (Sigma). Control samples were similarly treated in the absence of enzyme. 

### ELISA for soluble VE-cadherin detection in human serum

The assay is a sandwich enzyme immunoassay using a monoclonal antibody and an enzyme-linked polyclonal antibody specific for sVE-cadherin, as described in supplementary information (see Tables S1, S2, S3, and S4 and Figure S1 in Methods S1 file). The calculations were performed independently. Raw data (OD) measured by the plate reader were plotted against nominal standard concentrations to construct the standard calibration curves. Concentration values of unknown samples were interpolated from these curves using an unweighted linear regression of the data. A typical standard curve is presented in Figure S1D in Methods S1 file. Throughout the study, the percent of coefficients of variation (%CV) of standards were from 1.26 to 9.54%. The patients sera (5 µL) were diluted 1:100 dilution in 0.5% Triton X100-containing PBS. Individual serum concentrations for VE-cadherin are reported in ng/mL.

### Glioma Patients for Serum analysis

Brain tumor patients had blood sampling at their first radiological investigation. Fifty three patients diagnosed with a primary brain tumor glioma were classified by the following inclusion criteria: histopatologically proven oligodendroglioma, or mixed tumor; no history of brain tumor; magnetic resonance imaging (MRI) prior to any surgery (stereotactic biopsy or resection); no chemotherapy or radiotherapy prior to MRI, and survival time of at least 3 months after MRI. Data collected included standard demographics and disease characteristics; first date of treatment; best response to treatment and date of progression, date of death, or last follow up. Patients were treated with Temodal® alone, or by Temodal® with radiotherapy following the Stupp protocol [2]. Response to therapy was investigated using MRI following Cairncross criteria. After initial diagnosis, follow up was both clinical and radiological, systematically including a MRI control every 4 to 6 months. 

### Statistical analysis

#### For cell analysis and ex vivo analysis

All of the experiments were repeated at least three times. Values represent the mean ± standard deviation of three determinations from three different wells or dishes in the same experiment. All western blot bands have been subjected to densitometry; data are expressed in arbitrary units as the mean +/- SD of at least 3 identical experiments and were compared using Student’s *t*-test. For all tests, *P* values less than or equal to 0.05 were considered significant.

#### For glioma patients analysis

Patient characteristics data were summarized in terms of size and frequency for categorical data and by mean ± standard deviation for quantitative data. Independence between qualitative parameters was assessed using either the t-test or chi-square test. Taking into account the non-normal distribution of the sVE in the patient population data, the Mann-Whitney’s U-test, a non-parametric method, was conducted to compare sVE by death and survival groups. Survival time was defined as the time period between the initial radiological investigation including the blood collection and the date of death or last follow-up, taking into account that the follow-up period for surviving patients was at least 3 years at the end of the study (July 31^st^ 2009). Several clinical and MRI factors were tested for their prognostic value relating to survival time, including age, sex (male/female), tumor grade (II versus III-IV), contrast agent uptake (present) and sVE value. Univariate analyses were performed using Cox proportional hazard models and presented as Hazard Ratio with 95% confidence intervals. Overall survival curves were assessed using Kaplan-Meier’s method presented as a function of baseline sVE levels. Survival time was summarized by tercile group with 95% confidence intervals and compared using Cox model. For Cox model, proportional hazards assumption was validated on the basis of Schoenfeld residuals [21]. All data analyses were performed using Stata release 11.0 (StataCorp, College Station, TX) - PC software. P-values <0.05 were considered statistically significant. In figures, asterisks identify significantly different values. 

## Results

### VE-cadherin expression in glioblastoma tissue samples

**Figure 1:**
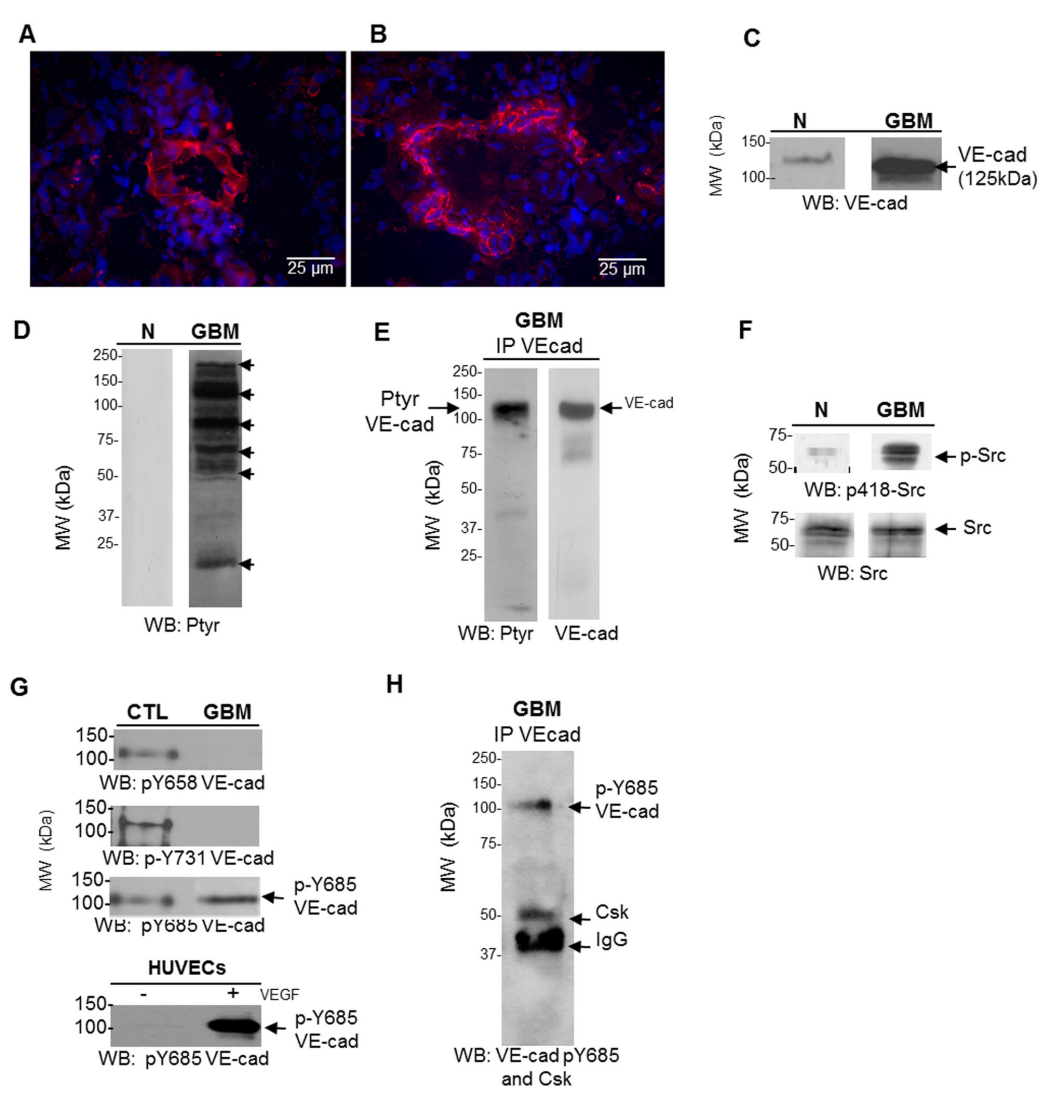
VE-cadherin expression and phosphorylation in human glioma tissues. (A,B) Representative (n=10 samples) micrographs of primary human glioma stained for VE-cadherin (in red; nuclei are stained in blue). Images were processed using Adobe Photoshop Scale bar: 25 μm. A capillary network positive for VE-cadherin was detected in all the tumors. (C) A 125 kDa fragment of VE-cadherin was highly detectable in glioblastoma (GBM) extract and not in non-tumor brain tissue (N). (D) Protein lysates from N and GBM were analyzed by SDS-PAGE and western blotting with the antiphophotyrosine antibody (clone 4G10). Several proteins with apparent molecular masses (indicated by filled arrowheads) ranging from 220 to 25 kDa displayed clearly strong tyrosine phosphorylation in GBM but not in non-tumor tissue. (E) 500 μg of GBM tissue lysate protein were immunoprecipitated with an anti-human VE-Cadherin antibody directed to C-term of the protein and blotted with the indicated antibodies (Ptyr or VE-cad). Immunoprecipitation of VE-cadherin from glioma extracts allowed to detect a tyrosine phosphorylated form of VE-cadherin. Sample loading was controlled using actin detection. (F) Active Src (phosphoY418) was highly detectable in GBM but not in non-tumor (N) brain extract. (G) Orthovanadate treated-HUVECs lysates (control: CTL) and GBM extracts (50 µg) were analyzed by western blot using antiphosphosite antibodies directed against Y^658^ and Y^731^, and the antibody directed against pY^685^ VE-cadherin raised in our laboratories. Only pY^685^ was detected in GBM as in HUVECs upon VEGF stimulation (50 ng/mL). (H) Same experiment as described in (E), and immunoblotting with anti-Csk antibody and anti-pY^685^ VE-cadherin antibody. The association of Csk with VE-cadherin in GBM confirmed the phosphorylation at the site Y^685^ also detected with the antiphosphosite antibody. Filled arrowhead indicates the position of the IgG heavy chains of the crosslinking antibody. In all blots the position of size markers (in kDa) is indicated on the left. These experiments were repeated at least three times in a similar configuration.

A first series of experiments were designed to visualize VE-cadherin expression in frozen tissue sections of human glioblastoma (GBM) by immunohistochemistry staining using an antibody raised against the VE-cadherin C-terminal domain. All the surgical resections exhibited a tumor vasculature, positive for VE-cadherin indicating that VE-cadherin represents a marker of glioma capillary network (Figure 1A and B (n=10), grade III-IV). VE-cadherin expression in human glioma tissue extracts were further confirmed by western blotting (Figure 1C). VE-cadherin is a 784 amino acids (AA) polypeptide whose cytoplasmic domain contains 164AA. As VE-cadherin is a glycosylated protein, in SDS-PAGE it migrates with an apparent molecular weight of 125 kDa. One major species of 125 kDa corresponding to VE-cadherin was detected in the brain tumor tissue, while the protein was barely detectable in non-tumor brain tissue (Figure 1C). This high expression level of VE-cadherin in malignant gliomas is consistent with their reported extensive neovascularization [22,23].

### Characterization of VE-cadherin tyrosine phosphorylation status in glioblastoma tissue

 Receptor tyrosine kinase (RTK) signaling pathways regulate many aspects of tumorigenesis, including cell growth and proliferation [24]. We thus examined the panel of tyrosine phosphorylated proteins in human non-tumor (N) and tumor brain (glioblastoma, GBM) tissues. As shown in Figure 1D, no phosphotyrosine containing proteins were detected in non-tumor brain tissue, while several proteins with apparent molecular weight ranging from 25 to 250 kDa clearly displayed enhanced tyrosine phosphorylation in tumor (filled arrowheads). To prove the existence of endogenous phosphorylation of VE-cadherin in GBM tissue, VE-cadherin immunoprecipitation was performed and its phosphorylation status was analyzed with a specific monoclonal antibody recognizing tyrosine phosphorylated residues (Figure 1E). Tyrosine-phosphorylated VE-cadherin was detected in all GBM tissue samples but not in non-tumor brain samples. This data is consistent with the abnormal activation of RTK signaling pathways reported in malignant glioma [25].

### Src family tyrosine kinase expression in human brain tumor tissues

As we have previously shown that VE-cadherin was a substrate for Src kinase [15], we next analyzed the activation status of Src kinase in brain tumor tissue extracts. Kinases of Src family are involved in VEGFR-2 signaling known to regulate vascular permeability, angiogenesis [26,27], cell motility and apoptosis [28]. Autophosphorylation at Y^418^ causes an increase in the activity of Src towards exogenous substrates. Phosphorylation at Y^527^ by C-terminal Src kinase (Csk) results in inhibition of Src tyrosine kinase activity [29,30]. As illustrated in Figure 1F, the activated form of Src (phosphoY^418^) was highly detectable in brain tumor extracts, while it was barely detectable in non-tumor brain. The enhanced Src kinase activity was also measured by exogenous substrate phosphorylation (data not shown). 

### VE-cadherin tyrosine phosphorylation at Y685 residue in human glioblastomas tissues

We have previously shown that VEGF induced VE-cadherin tyrosine phosphorylation at site Y^685^ upon Src kinase activation [15]. Other VE-cadherin tyrosine phosphorylation sites have been reported elsewhere, including Y^658^ and Y^731^ sites which are involved in increased permeability *in vitro* [31]. As VE-cadherin tyrosine phosphorylation sites have never been explored in human tumor tissues, we analyzed phospho-VE-cadherin tyrosine sites in GBM extracts, using two commercial anti-VE-cadherin phosphosites directed against sites Y^658^ and Y^731^ and an antibody anti-phosphoY^685^ raised in our laboratories. The phosphosites Y^658^ and Y^731^ were not detected in human glioblastoma tissue extracts (Figure 1G, GBM), while they were in HUVECs (Figure 1G, CTL). Using antibody raised in our laboratories that recognized phospho-VE-cadherin in VEGF-stimulated HUVECs (positive control; Figure 1G-HUVECs), phosphorylation on Y^685^ VE-cadherin was detected in all human glioblastoma tissues tested in this study (Figure 1G, GBM). The phosphorylated Y^685^ was reported to be a binding site for the SH2 domain of the tyrosine kinase Csk, a negative regulator of Src [32]. Thus, to further confirm the presence of phosphorylation on pY^685^, VE-cadherin was immunoprecipitated from human GBM extracts and probed with anti-Csk and anti-phospho-Y^685^VE-cadherin antibodies. As shown in Figure 1H, Csk was found to be associated with VE-cadherin immunoprecipitates and VE-cadherin was phosphorylated on Y^685^. Altogether, these results for the first time provide evidence for VE-cadherin tyrosine phosphorylation in human brain tumors and at a tyrosine site previously identified in the VEGF signaling pathway, which is consistent with the high expression of VEGF in malignant glioma [33].

### VEGF induced-VE-cadherin tyrosine phosphorylation preceded N-terminal ectodomain shedding of the protein

**Figure 2:**
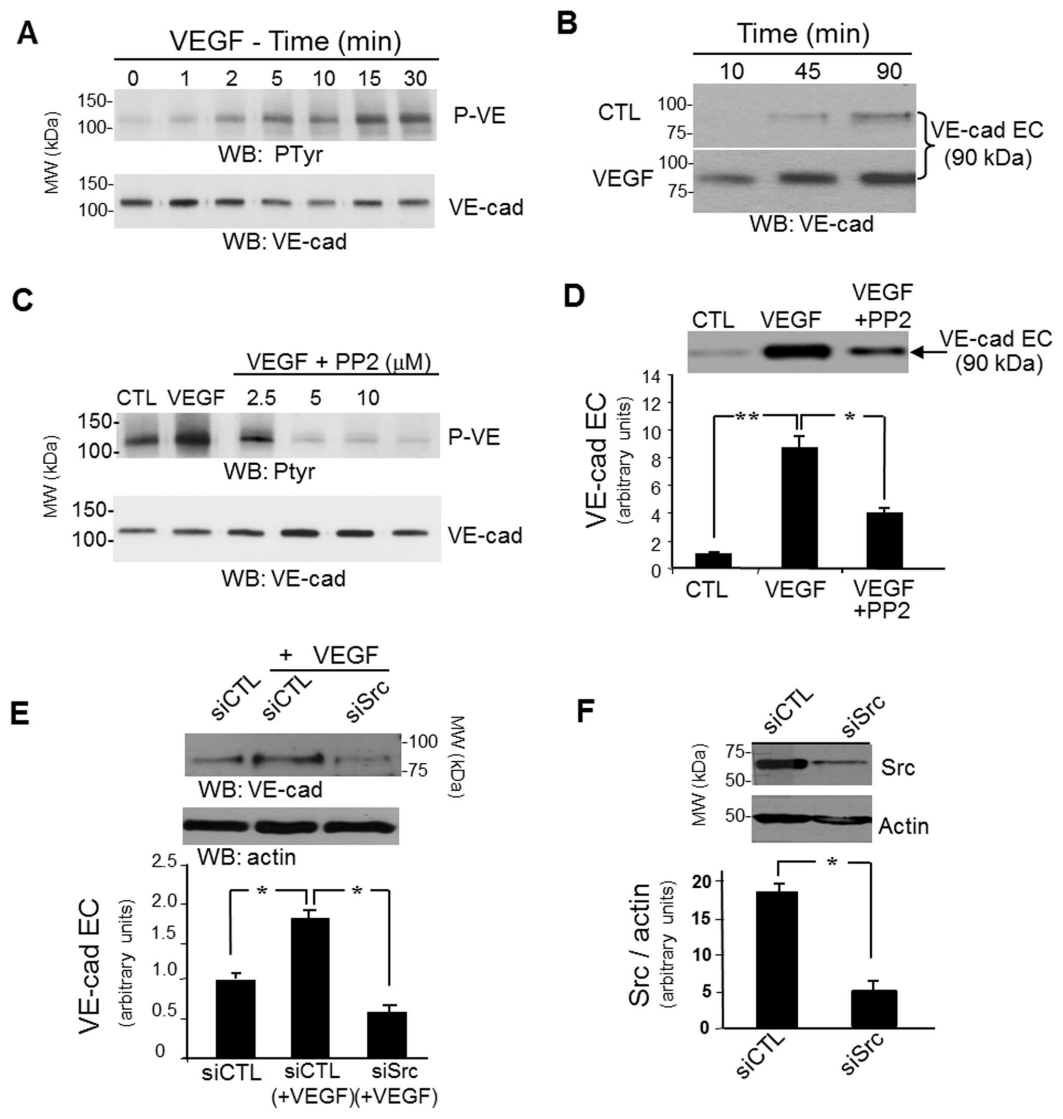
VEGF induced-VE-cadherin extracellular domain cleavage is preceded by a Src-dependent VE-cadherin tyrosine phosphorylation. (A,B) HUVECs treated with VEGF (50 ng/mL) were analyzed for phosphotyrosinated-VE-cadherin in cell extracts (A) and VE-cadherin extracellular domain in conditioned medium (B): (A) VE-cadherin was immunoprecipitated from 200 µg of protein lysates and analyzed by SDS-PAGE and western blotting with the anti-phosphotyrosine antibody. VEGF induced a time-dependent tyrosine phosphorylation of VE-cadherin detectable after 2 min of stimulation. (B) Conditioned media from VEGF-stimulated HUVECs were concentrated and analyzed by SDS-PAGE and western blotting with human VE-cadherin antibody directed against VE-cadherin extracellular domain (BV9). A 90 kDa fragment corresponding to the full length VE-cadherin extracellular domain was already detectable after 10 min of VEGF stimulation. (C) HUVECs were pretreated with increasing concentrations of Src inhibitor PP2 (2.5 to 20 µM) for 15 min, prior to treatment with VEGF for 15 min. VE-cadherin was immunoprecipitated from 200 µg of protein lysates. PP2 concentrations higher than 2.5 µM completely inhibited VEGF-induced VE-cadherin tyrosine phosphorylation. (D) Analysis of conditioned media from an identical number of HUVECs pre-treated for 15 min with PP2 (5 µM) before VEGF stimulation for 15 min: the inhibitor decreased VEGF-induced VE-cadherin cleavage. (E,F) Src expression was inhibited by Src-siRNA in HUVECs 24 hours before VEGF stimulation (15min). Analysis of conditioned media showed that the knock-down of Src (controlled in F) decreased the level of soluble VE-cadherin in the media. (D,E,F) The signals were quantified using ImageJ software, error bars in graphs indicate S.D. and experiments were repeated at least three times in a similar configuration.

Because VEGF is a major compound of tumor microenvironment, involved in tumor angiogenesis, tumor aggressiveness and disease prognosis in patients [34], we next examined whether VEGF was involved in VE-cadherin ectodomain shedding in a concomitant manner with tyrosine phosphorylation of the protein. To that purpose, confluent HUVECs monolayer was submitted to VEGF challenge (50 ng/mL) in a kinetic study for up to 45 min. The culture media were collected and analyzed by SDS-PAGE and western blotting for soluble VE-cadherin content. In parallel, the HUVECs monolayer was scrapped and lysed for further analysis of VE-cadherin tyrosine phosphorylation status by immunoprecipitation. As shown in Figure 2A, the exposure of the endothelial cells to VEGF induced an early VE-cadherin tyrosine phosphorylation detected within 1-2 min of stimulation which increased up to 30 min. Analysis of cells conditioned medium showed that a 90 kDa immunoreactive fragment (sVE) corresponding to the full ectodomain of the protein was detectable within 10 min of VEGF stimulation and increased in a time-dependent manner up to 90 min (Figure 2B). This result suggests that VE-cadherin tyrosine phosphorylation in its cytoplasmic domain preceded the cleavage of extracellular domain probably by increasing VE-cadherin susceptibility to proteolysis. Consistent with this, HUVECs pretreatment with the Src family kinase inhibitor PP2 prior to VEGF stimulation decreased VEGF induce VE-cadherin phosphorylation (Figure 2 C) and cleavage (Figure 2 D). To confirm the specific function of Src in this process, we knocked Src expression down in HUVECs using cSrc-specific siRNA (siSrc) which efficiently impaired VEGF-induced sVE release in cell medium (Figure 2 E), while control siRNA (si-CTL) had no effect (Figure 2 F). This result demonstrates that VEGF-induced cleavage of VE-cadherin adhesive ectodomain is Src-dependent, suggesting a similar pathway to the previously observed effect of TNFα and confirmed a critical role for Src in this process [17].

### Matrix Metalloproteinases (MMPs) from Glioma cell lines induced VE-cadherin extracellular domain cleavage

**Figure 3:**
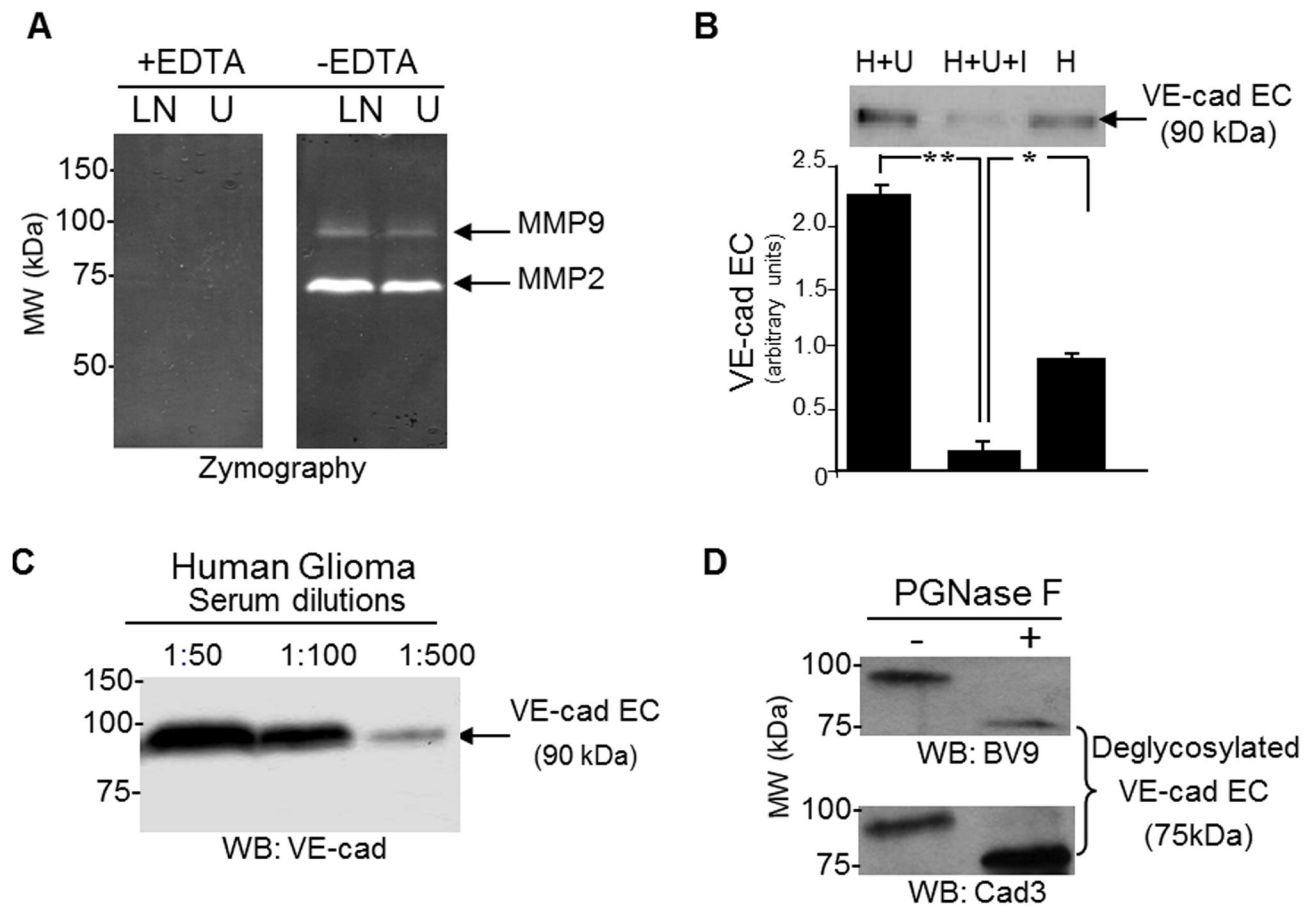
Metalloproteinases are secreted by glioma cell line and induced VE-cadherin cleavage. (A) Conditioned media from Astrocytoma grade IV (LN229, LN) and Astrocytoma grade III (U87, U) cells lines were tested for protease activities using a zymography assay. Inhibition of protease activities by EDTA identified MMPs. (B) U87 (U) cell line media induced VE-cadherin cleavage from HUVECs (90 kDa fragment). Glioma cell line conditioned media was added to HUVEC confluent monolayer during two hours and HUVEC (H) conditioned media was analyzed for sVE content by western blot. The effect was impaired by broad spectrum MMPs inhibitor (GM6001, I). (C) Western blot analysis of glioma patient sera at dilution 1:50, 1:100, 1:500 revealed the presence of the 90 kDa fragment of VE-cadherin (sVE). (D) Deglycosylation Assay of sVE in serum shows, using two different antibodies to sVE that the soluble fragment is glycosylated.

 Several MMPs have been implicated in modifying the extracellular matrix (ECM) in the tumor microenvironment to promote invasion [35]. We thus examined whether glioma cell lines secreted MMPs and whether these proteases could induce VE-cadherin cleavage. Using the zymography technique, we analyzed the presence of gelatinase activities in the conditioned media from two glioma cell lines, LN229 and U87 (LN, U). Two gelatinase activities were detected and their activity was abolished in the presence of EDTA, suggesting that these enzymes belong to the MMP family (Figure 3A). Based on their molecular weight, we concluded that MMP-2 and MMP-9 were present in the conditioned media under their activated forms. This result is in agreement with the previously demonstrated invasive potential of glioma with increased extracellular matrix disruption in part by the action of MMPs [35,36]. When conditioned media from LN229 cell line was applied onto HUVECs monolayers (H), the release of the sVE was observed (H+L). This effect was blocked by GM6001 pretreatment (I), a potent inhibitor of MMP activities, demonstrating the involvement of these proteinase activities in VE-cadherin extracellular domain cleavage (Figure 3B). These findings, together with the previous demonstration of VE-cadherin tyrosine phosphorylation in tumors, suggest that such modifications of VE-cadherin might occur in response to tumor microenvironment in human brain tumors.

### Soluble VE-cadherin (sVE) was detected in glioma patient serum

To determine whether VE-cadherin cleavage would also occur in glioma patients, we first analyzed a serum obtained from a glioma patient by SDS-PAGE and western blotting. A single soluble 90 kDa fragment of VE-cadherin (sVE) was detected (Figure 3C). This soluble fragment was found to be glycosylated as PGNase treatment of the serum prior to SDS-PAGE induced a shift in the apparent protein molecular weight (Figure 3D). This data is consistent with the presence of several glycosylation sites in the extracellular domain of VE-cadherin [37]. To quantify sVE in patient sera, we could not use commercially available kit because the standard used for calibration curve was not glycosylated. We thus designed an ELISA to quantify sVE in glioma patient sera using a recombinant human glycosylated VE-cadherin of 90 kDa as a standard for the internal calibration curve (See Figure S1 in Methods S1 file).

### Pathological and clinical data

sVE was quantified in a cohort of 53 glioma patients from Grenoble University Hospital. We investigated whether the levels of sVE measured in newly diagnosed glioma patients and the clinical features were associated with the overall survival from the time of initial diagnosis. The time to progression or patient death was deﬁned as the time elapsed between the date of the initial radiological investigation and baseline blood draw, and the date of clinical progression or death or, if neither progression nor death was observed during the follow-up period, the date of the last follow-up visit. The follow-up period for surviving patients was up to 3 years (that is 1,095 days) at the end of the study (July 31^st^ 2009). Baseline characteristics of all brain tumor patients included in this study are summarized in Table 1 (n=53).

**Table 1:** Description of patients characteristics (n=53) at initial diagnosis.

**Characteristic**		**Value**
**Age**	Median – year	48
	Range – year	19-73
	<65 year – no. (%)	47 (88)
**Sexe – no. (%)**	Male	34 (64)
	Female	19 (36)
**Diagnosis – no. (%)**	Astrocytoma	25 (47)
	Oligodendroglioma	26 (49)
	Nd	2 (4)
**WHO classification at initial diagnosis – no. (%)**		
**Astrocytoma**	All	25 (47)
	Grade II	7 (11)
	Grade III	2 (4)
	Grade IV	15 (28)
**Oligodendroglioma**	All	26 (49)
	Grade II	18 (34)
	Grade III	8 (15)
	Nd	2 (4)

### Description of patient outcome at 3 years

**Figure 4:**
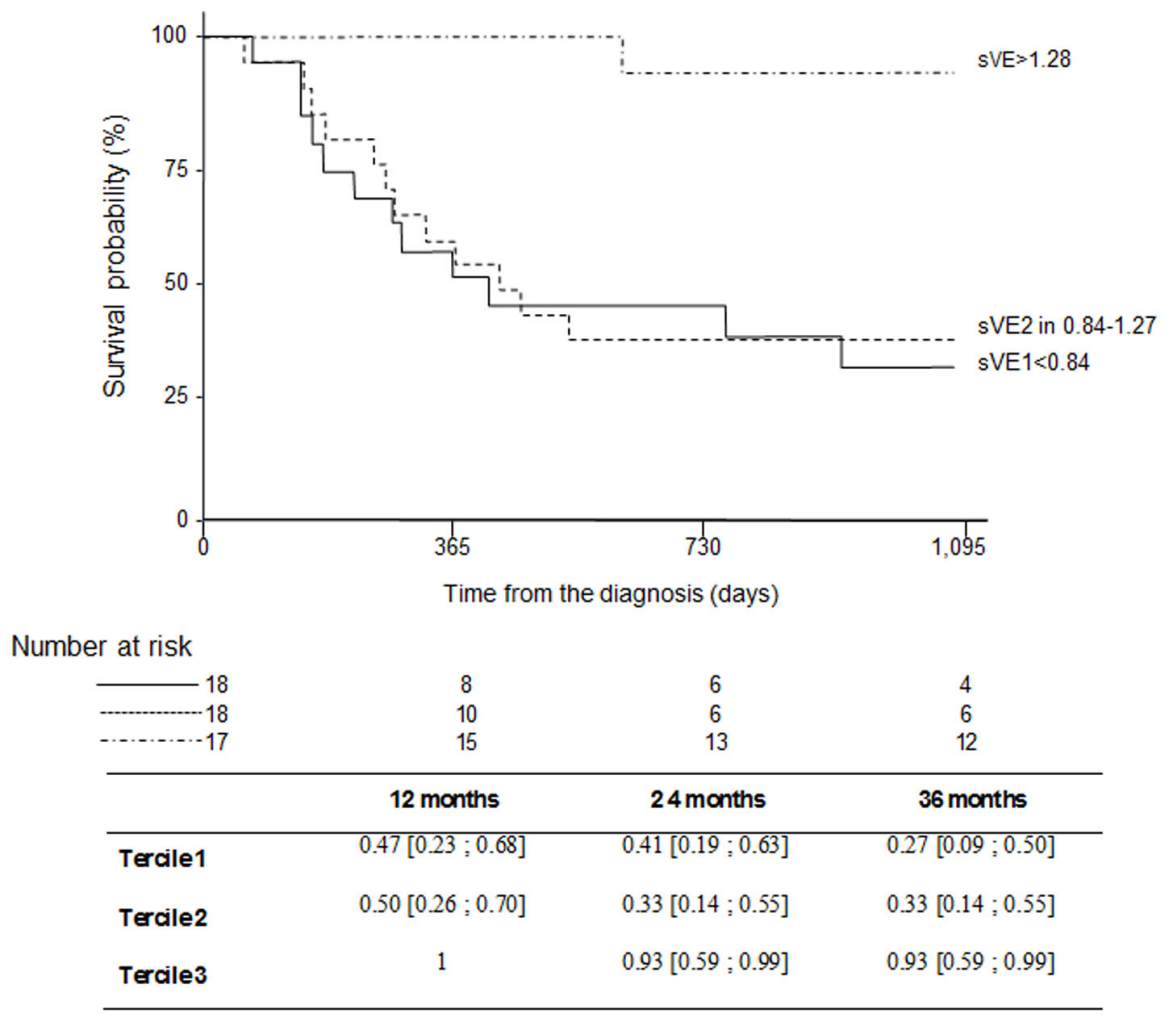
Kaplan-Meier estimates overall survival at 3 years according to baseline soluble VE-cadherin. Patients with sVE< 840 ng/ml had a median time to progression of 220 days. Patients with 840 < sVE <1,247 ng/ml had a median time to progression of 365 days whereas patients with sVE >1,247 ng/ml had a median time to progression of three years, irrespective to histopathological grade of tumors.

**Figure 5:**
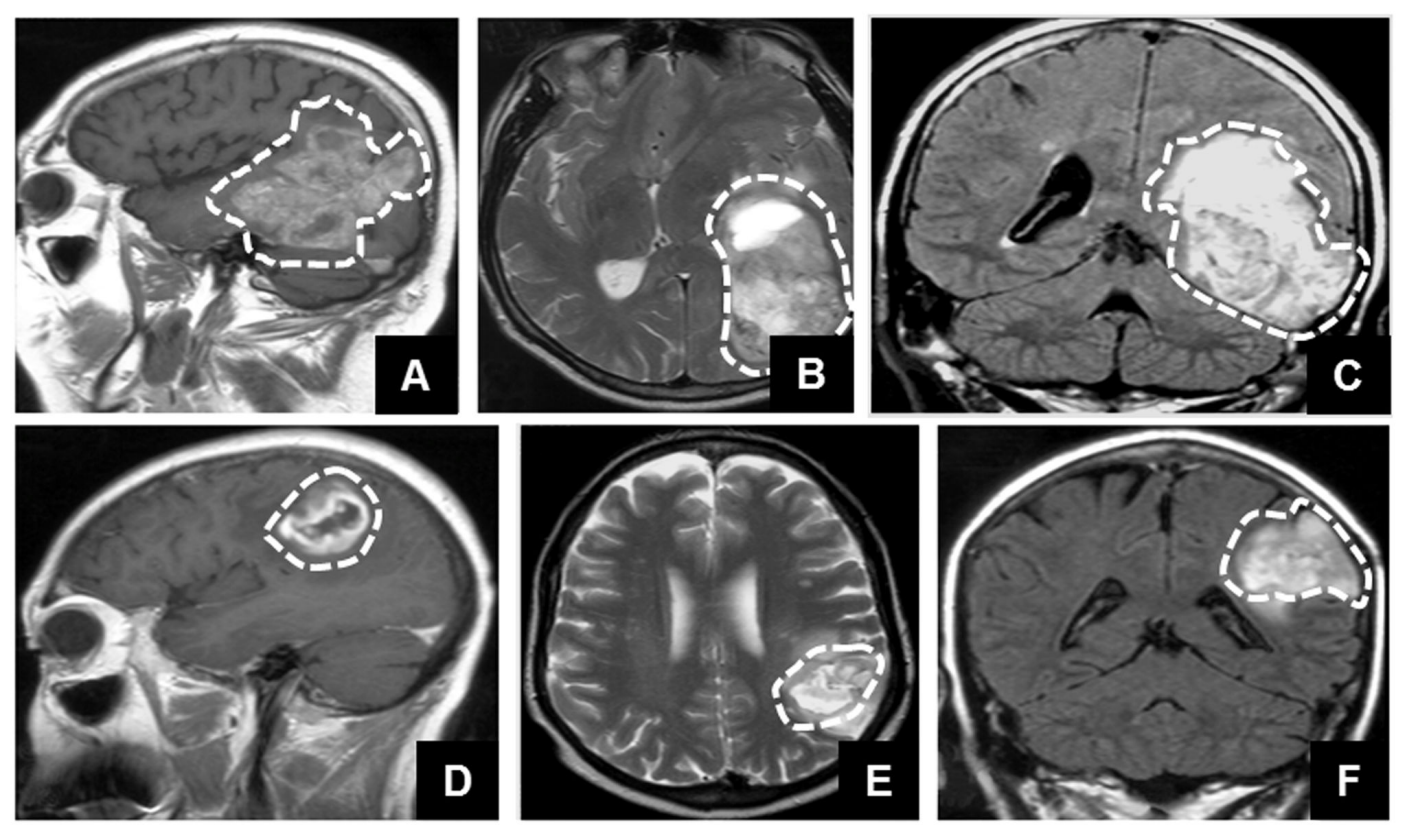
Illustrative cases. All MRI examinations were performed using a 1.5 Tesla scanner (Philips Medical System). Standard MRI work-up systematically comprised at least one series of T2-weighted images (turbo spin echo, repetition time msec (RT)/echo time msec (ET=0.625/120; numbers of signals averaged ((NAS)=2; turbo factor=15) and T1-weigthed images (spin echo, RT/ET= 500/10; NAS=2) obtained prior to and after gadolinium injection. Representative contrast-enhanced images from low (a,b,c) and high (d,e,f) levels of sVE patients. (A,B,C) 36 years old man, oligodendroglioma grade III, in the left posterior temporal region (6 cm major axis): (A) Sagittal T1-weighted image after gadolinium injection shows diffuse and extensive contrast enhancement, (B) axial T2-weighted image shows heterogeneous aspect and few perilesional edema of the same lesion with (C) T2/ﬂuid attenuated inversion recovery (FLAIR) shows infiltrative lesion with mass effect on ventricular junction. On all panels, the tumor area is indicated using a dotted white line. For this patient, sVE=296 ng/mL and overall survival was 12 months. (D,E,F) 60 years old women, glioblastoma in the left parietal region (3.5 cm major axis): (D) Sagittal T1-weighted image after gadolinium injection shows a ring of contrast enhancement around an area of hypointensity (necrosis). (E) axial T2-weighted image (T2) shows irregular contours and significative perilesional edema. (F) T2/ﬂuid attenuated inversion recovery (FLAIR) shows few mass effect. For this patient, sVE=1.843 µg/mL and overall survival was 36 months.

Of the 53 patients analyzed, 28 were still alive at the end of the study (July 31^st^ 2009), all presenting survival time of 1095 days. The median survival was 930 days for the entire group. Tumors were classified after histological analysis according to the WHO classification [3], as grade II (n=25; 47.2%), grade III-IV (n= 28; 52.8%). The average size of the tumors was 44.3 ± 15.3 mm with variable locations. Conventional imaging (MRI) was performed in conjunction with physical examination at regular time intervals for all patients throughout the study to monitor disease status. In MRI, contrast agent uptake by the tumor was present in 34 patients (64%), and absent for 19 patients (36%), images were not recorded for 2 patients. With respect to clinical findings, prognostic factors identified in univariate analysis are listed in Table 2. These factors are age (p=0.002), sex (p=0.0260), tumor grade (p<0.001), contrast uptake by the tumor (p=0.008) and sVE (p<0.001). In the entire population studied here, the baseline sVE level varied from 236 to 2,000 ng/ml. Stratification of the patient population according to sVE is presented as Kaplan–Meier curves in Figure 4. P values from univariate Cox modelindicate that sVE was significantly predictive of the overall survival (HR 0.13 [0.04;0.40] P≤0.001). Patients with sVE< 840 ng/ml had a median time to progression of 220 days. Patients with 840 < sVE <1,247 ng/ml had a median time to progression of 365 days whereas patients with sVE >1,247 ng/ml had a median time to progression of three years, irrespective to histopathological grade of tumors. Of major interest, the low sVE level was associated with a shorter time to progression. Several series of MRI images were acquired for all the patients before starting the treatment. Two illustrative cases with opposite sVE concentrations are presented in Figure 5. The low sVE (296 ng/mL, overall survival: 12 months) patient (A,B,C) was a 36 years old man presenting an oligodendroglioma grade III in the left posterior temporal region (6 cm major axis): This tumor presented diffuse and extensive contrast enhancement (A) and few perilesional edema (B), and was infiltrative with mass effect on ventricular junction (C). The high sVE (1.843 ng/mL, overall survival: 36 months) patient (D, E, F) was a 60 years old women presenting a glioblastoma in the left parietal region (3.5 cm major axis): This tumor presented a ring of contrast enhancement around an area of hypointensity (necrosis) (D), with irregular contours and significative perilesional edema (T2, E), and few mass effect (F). 

**Table 2:** Prognostic factors and clinical outcome at 3 years.

**Prognostic factors**		**All patients**	**Death**	**Survival**	**P value**
		n=53	n=25	n=28	
**Age (year)**		48 ± 13.5	53.9 ± 11	42.6 ± 13	**0.002** ^(1)^
**Sex**	Male	34 (64%)	18 (52%)	16 (47%)	0.260 ^(3)^
	Female	19 (35%)	17 (36%)	12 (63%)	
	Grade II	25 (47%)	5 (20%)	20 (32%)	**0.001** ^(3)^
**Histology**	Grade III	13 (24%)	8 (61%)	5 (38%)	0.232 ^(3)^
	Grade IV	15 (28%)	12 (80%)	3 (20%)	**0.003** ^(3)^
**Contrast enhancement**					
(present)		32 (62%)	18 (56%)	14 (43%)	0.088 ^(3)^
**sVE (µg/mL)**		1.06 ± 0.40	0.85 ± 0.31	1.24 ± 0.39	**0.001 ^(2)^**

t-test ^(1)^ ; Mann-Whitney’s U-test ^(2)^; Chi^2^ test ^(3)^.

## Discussion

 Angiogenesis in brain tumors has been extensively characterized over the last two decades using several experimental approaches including extensive genomic, transcriptomic and proteomic screening [7,38,39]. Nevertheless, VE-cadherin, which represents a specific endothelial marker, has never been explored in terms of post-translational modifications in this context. We demonstrate for the first time the strong expression of the protein in human glioma tissues, and its phosphorylation on tyrosine Y^685^ that might represent the identity card of Src action upon VEGF secreted by tumor cells. Finding this site in human brain tumors might reflect a major role for phospho-Y^685^ in angiogenesis and/or permeability driven by VEGF as Src was also reported to be involved in VEGF-induced permeability [26]. 

In addition to active Src in these tumors, we observed high level of tyrosine-phosphorylated proteins in agreement with the abnormal activation of multiple RTK signaling pathways in glioblastoma [40]. It was recently shown that coexpression of phosphorylated Dock180 (Y^1811^), phosphorylated Src (Y^418^) and PDGFRα was predictive for extremely poor prognosis in patients with gliomas [41]. As we have analyzed human samples prior patient treatment, and because the baseline characteristic of the tumors indicate a high level of tyrosine-phosphorylated proteins including VE-cadherin, this data also re-inforced the interest for tyrosine kinase inhibitors in therapy [42]. Genomic approaches applied to clinically characterized patient cohorts now clearly show that combined molecular and histological classification offers great opportunities to significantly improve clinical predictive power over the use of histology only [43]. Currently, three molecular markers, related to a better outcome, are particularly useful and complement the histological classification: the 1p/19q codeletion, the O(6)-methylguanine-DNA methyltransferase promoter methylation and the substitutions in the isocitrate dehydrogenase (IDH1) gene [44]. Further work is needed to assess whether phosphoY^685^VE-cadherin can be considered as a tissue marker of angiogenic activated capillary network in human glioma correlated with histological grade in order to improve histological classification of brain tumors. 

**Figure 6:**
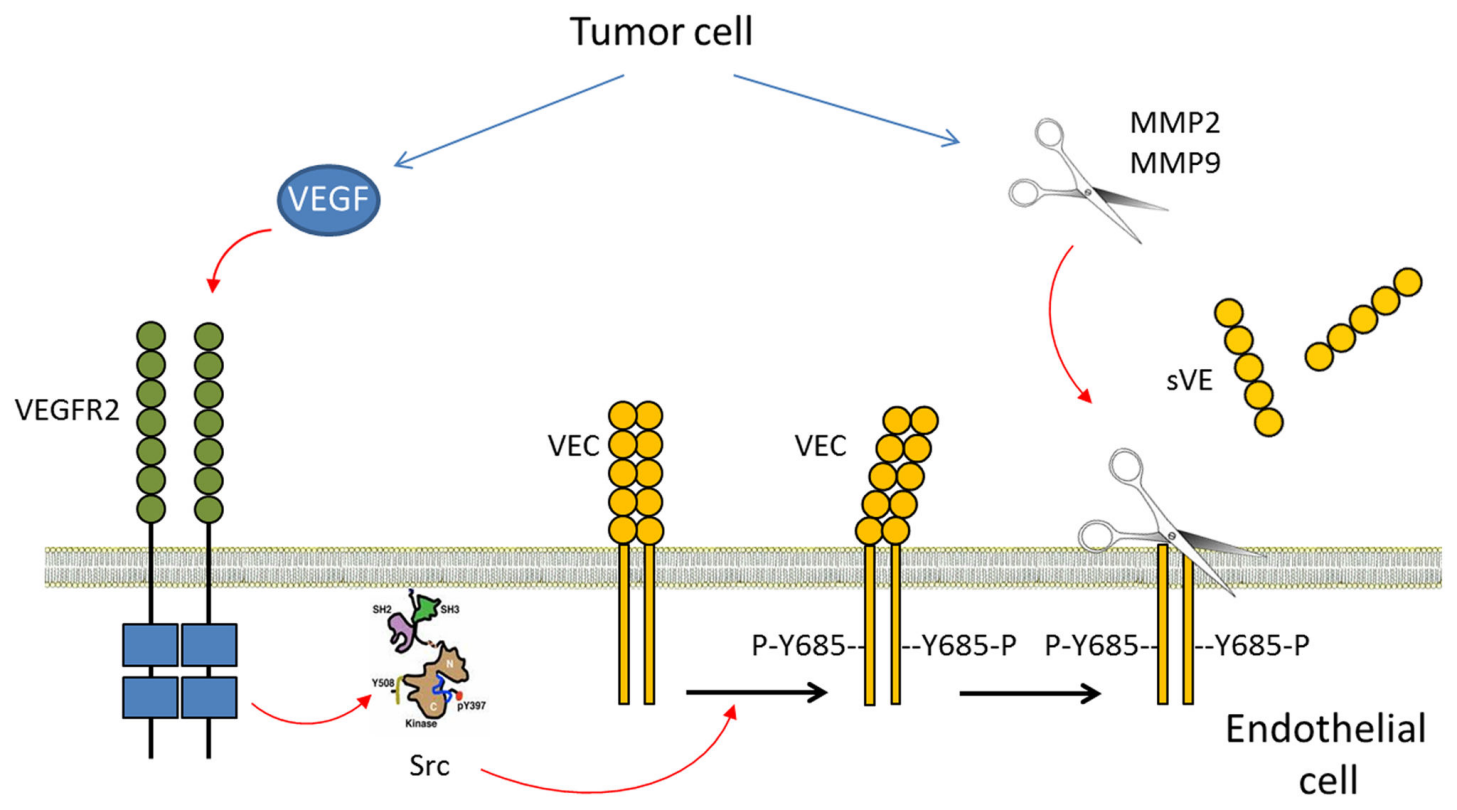
Scheme for hypothetical mechanism of a link between VE-cadherin phosphorylation and cleavage. In tumours, VEGF secreted by tumoral cells bind to VEGFR2 which leads to src activation. Activated src rapidly phosphorylates VE-cadherin on Y685. This covalent modification of the protein is followed by the cleavage of its extracellular domain upon MMP2,9.

We demonstrated that compounds of the tumor microenvironment, such as VEGF and MMPs secreted by glioma cell lines, can induce VE-cadherin extracellular domain cleavage, which is consistent with the involvement of the stroma and associated matrix proteins in cancer cell invasion and proliferation [45]. For VEGF, the mechanism involves Src kinase activity, confirming the requirement of this tyrosine kinase in VE-cadherin cleavage as we previously reported for TNFα, and then indicating a common pathway for both cytokines. The kinetic study of VEGF-induced sVE shedding showed that sVE became detectable in cell media 10 min after VE-cadherin tyrosine phosphorylation in its cytoplasmic tail. This result strongly suggests that VE-cadherin tyrosine phosphorylation is required for the cleavage of its extracellular domain. Thus, activation of Src in response to VEGF in the brain tumors is a potential mechanism that could explain the presence of soluble VE-cadherin in the blood from glioma patients (Figure 6). On the other hand, glioma cells secrete MMPs which might also act on glioma vasculature, thus participating in the release of sVE in blood, and then partly account for highly aberrant, tortuous and hyper-permeable intratumor vasculature [5,46]. In addition, such abnormal endothelial cells from these vessels might also secrete MMPs in response to VEGF as already observed [47]. MMPs from both origins might be involved in VE-cadherin cleavage. Several reports demonstrated that changes in cerebrovascular parameter measurement are of importance in the diagnosis and the follow-up for glioma patients [48–50]. Indeed, the techniques used for that measurement are based on the modification of permeability of the blood brain barrier and gadolinium-diethylenetriamine pentaacetic acid extravasation. It remains to be established whether sVE is correlated with analysis of magnetic resonance perfusion images, and whether it could be a potential parameter in addition to size, mean of relative cerebral blood volume (rCBV), mean of leakage coefficient and hyperperfusion volume, to optimize rCVB maps in predicting glioma grade. In addition, because VEGF induced VE-cadherin cleavage, sVE levels quantification might be an interesting companion tool for MRI to follow the patient responsiveness to antiangiogenic therapies, as MRI assessments may be misleading in that potent anti-VEGF agents can decrease permeability and may lessen contrast enhancement with or without a true underlying antitumor effect.

 Interestingly, we described for the first time the presence of sVE (90 kDa) in sera from glioma patients. The western blot analysis technique demonstrated that sVE in serum exhibited the same molecular weight as sVE found in EC upon VEGF challenge, and that the sVE found was glycosylated. The design of a new ELISA using the human recombinant glycosylated VE-cadherin extracellular domain as calibration standard allowed an easy quantification of sVE in glioma patient sera prior treatment. As the commercially available kit displayed the non-glycosylated VE-cadherin recombinant for the standard curve, it could not be used in this study due to the potential differences in antibodies affinities. The analysis of the glioma cohort demonstrated a correlation between baseline sVE level and overall survival (p<0.001) independently of histological grade. The low sVE level was associated with a shorter time to progression indicating that the most refractory patients to therapies had low levels of sVE. Consistent with this, Sitohy et al. reported recently that the tumor blood vessel heterogeneity is accountable for the difference between vessel subpopulations in their requirements for tumor cell–secreted VEGF [50]. The most resistant tumors to therapies in their model presented vessels with low levels of VEGFR2. In the same way, patients who were most refractory to therapies may have some major tumor vessels with low level of VEGFR2, and thus explanation of the low level of sVE as VEGFR2 is required for VEGF signal transduction. Even if the incidence of brain tumors is lower than other cancers, a larger prospective series of patients with malignant brain tumor is needed to further explore sVE. 

During the last decade, many proteomics studies, using mass spectrometry-assisted analysis of tissue or blood, reported protein fragments as potential biomarkers associated with a large panel of cancerous pathologies [51]. The protein cleavage process was rarely investigated and these fragments were usually not validated and did not move from bench to bedside. Our dual experimental and bioclinical approach allowed us to associate biomarker candidates (comprising a systemic one) and a potential responsible molecular mechanism that reinforces robustness of our findings. 

The key role of VEGF in tumor progression has been largely demonstrated for several decades. Unexpectedely, large discrepancies concerning VEGF as a potential biomarker did not allow the clinical validation of its quantification. Indeed, VEGF has a high affinity for extracellular matrix components and can be stored in tumor microenvironment. In addition, secreted VEGF can bind to its soluble receptors leading to a decrease in its bioavailability and modifiy quantification [52]. Furthermore, during blood sampling platelets can release VEGF in serum, leading to an overestimation of its concentration. Thus, these phenomenons prevent reliable evaluation of soluble VEGF produced by the tumor. Considering VE-Cadherin, as it is a specific component of the endothelial cells and because it has not been reported to be traped by ECM, soluble VE-cadherin in blood might reflect VEGF activity at tumor site. 

Taken together, these results indicate that VE-cadherin, a protein exclusively expressed in endothelial cells, is subjected to structural modifications in the tumor microenvironment. These modifications should be examined as candidate biomarkers in brain tumors because of the major roles of this protein in angiogenesis as well as in vascular permeability. Indeed, similar data were obtained in several diseases associated with vascular disorders (hereditary angioedema, rheumatoid arthritis). Thus, in further studies sVE which has a prognosis value might be associated with typical clinical or biological information to improve patient clinical management.. 

## Supporting Information

Methods S1 (DOCX)

## References

[1] Wrensch M, Minn Y, Chew T, Bondy M, Berger MS (2002) Epidemiology of primary brain tumors: current concepts and review of the literature. Neuro-Oncology 4: 278–299. doi:10.1093/neuonc/4.4.278. PubMed: 12356358. 12356358 PMC1920665

[2] Stupp R, Mason WP, van den Bent MJ, Weller M, Fisher B et al. (2005) Radiotherapy plus concomitant and adjuvant temozolomide for glioblastoma. N Engl J Med 352: 987–996. doi:10.1056/NEJMoa043330. PubMed: 15758009. 15758009

[3] Louis DN, Ohgaki H, Wiestler OD, Cavenee WK, Burger PC et al. (2007) The 2007 WHO classification of tumours of the central nervous system. Acta Neuropathol 114: 97–109. doi:10.1007/s00401-007-0243-4. PubMed: 17618441. 17618441 PMC1929165

[4] Carmeliet P, Jain RK (2011) Molecular mechanisms and clinical applications of angiogenesis. Nature 473: 298–307. doi:10.1038/nature10144. PubMed: 21593862. 21593862 PMC4049445

[5] Long DM (1970) Capillary ultrastructure and the blood-brain barrier in human malignant brain tumors. J Neurosurg 32: 127–144. doi:10.3171/jns.1970.32.2.0127. PubMed: 5411991. 5411991

[6] Gerstner ER, Sorensen AG, Jain RK, Batchelor TT (2008) Advances in neuroimaging techniques for the evaluation of tumor growth, vascular permeability, and angiogenesis in gliomas. Curr Opin Neurol 21: 728–735. doi:10.1097/WCO.0b013e328318402a. PubMed: 18989120. 18989120

[7] Ducray F, de Reyniès A, Chinot O, Idbaih A, Figarella-Branger D et al. (2010) An ANOCEF genomic and transcriptomic microarray study of the response to radiotherapy or to alkylating first-line chemotherapy in glioblastoma patients. Mol Cancer 9: 234. doi:10.1186/1476-4598-9-234. PubMed: 20822523. 20822523 PMC2944185

[8] Corada M, Mariotti M, Thurston G, Smith K, Kunkel R et al. (1999) Vascular endothelial-cadherin is an important determinant of microvascular integrity in vivo. Proc Natl Acad Sci U S A 96: 9815–9820. doi:10.1073/pnas.96.17.9815. PubMed: 10449777. 10449777 PMC22293

[9] Corada M, Zanetta L, Orsenigo F, Breviario F, Lampugnani MG et al. (2002) A monoclonal antibody to vascular endothelial-cadherin inhibits tumor angiogenesis without side effects on endothelial permeability. Blood 100: 905–911. doi:10.1182/blood.V100.3.905. PubMed: 12130501. 12130501

[10] Esser S, Lampugnani MG, Corada M, Dejana E, Risau W (1998) Vascular endothelial growth factor induces VE-cadherin tyrosine phosphorylation in endothelial cells. J Cell Sci 111 ( 13): 1853–1865. PubMed: 9625748. 9625748 10.1242/jcs.111.13.1853

[11] Dejana E, Orsenigo F, Lampugnani MG (2008) The role of adherens junctions and VE-cadherin in the control of vascular permeability. J Cell Sci 121: 2115–2122. doi:10.1242/jcs.017897. PubMed: 18565824. 18565824

[12] Hudry-Clergeon H, Stengel D, Ninio E, Vilgrain I (2005) Platelet-activating factor increases VE-cadherin tyrosine phosphorylation in mouse endothelial cells and its association with the PtdIns3’-kinase. FASEB J 19: 512–520. doi:10.1096/fj.04-2202com. PubMed: 15791001. 15791001 PMC4848345

[13] Lambeng N, Wallez Y, Rampon C, Cand F, Christé G et al. (2005) Vascular endothelial-cadherin tyrosine phosphorylation in angiogenic and quiescent adult tissues. Circ Res 96: 384–391. doi:10.1161/01.RES.0000156652.99586.9f. PubMed: 15662029. 15662029 PMC2798002

[14] fraser HM (2007) Regulation of angiogenesis in the ovary. Angiogenesis in endocrine tissues. Trivandrum. India: Vilgrain I. pp. 73–97

[15] Wallez Y, Cand F, Cruzalegui F, Wernstedt C, Souchelnytskyi S et al. (2007) Src kinase phosphorylates vascular endothelial-cadherin in response to vascular endothelial growth factor: identification of tyrosine 685 as the unique target site. Oncogene 26: 1067–1077. doi:10.1038/sj.onc.1209855. PubMed: 16909109. 16909109

[16] Bouillet L, Mannic T, Arboleas M, Subileau M, Massot C et al. (2011) Hereditary angioedema: key role for kallikrein and bradykinin in vascular endothelial-cadherin cleavage and edema formation. J Allergy Clin Immunol 128: 232–234. doi:10.1016/j.jaci.2011.02.017. PubMed: 21439626. 21439626

[17] Sidibé A, Mannic T, Arboleas M, Subileau M, Gulino-Debrac D et al. (2012) Soluble VE-cadherin in rheumatoid arthritis patients correlates with disease activity: evidence for tumor necrosis factor α-induced VE-cadherin cleavage. Arthritis Rheum 64: 77–87. doi:10.1002/art.33336. PubMed: 21905018. 21905018

[18] Baluk P, Hashizume H, McDonald DM (2005) Cellular abnormalities of blood vessels as targets in cancer. Curr Opin Genet Dev 15: 102–111. doi:10.1016/j.gde.2004.12.005. PubMed: 15661540. 15661540

[19] Hermant B, Bibert S, Concord E, Dublet B, Weidenhaupt M et al. (2003) Identification of proteases involved in the proteolysis of vascular endothelium cadherin during neutrophil transmigration. J Biol Chem 278: 14002–14012. doi:10.1074/jbc.M300351200. PubMed: 12584200. 12584200

[20] Identification of membrane calcium channels e... [ Growth Factors. 2001] -——.NCBI (n.d.). Available: http://www.ncbi.nlm.nih.gov/pubmed/11678208. Accessed 24 June 2013 10.3109/08977190109001074 11678208

[21] Schoenfeld D (1982) Partial residuals for the proportional hazards regression model. Biometrika: 239–241.

[22] Kargiotis O, Rao JS, Kyritsis AP (2006) Mechanisms of angiogenesis in gliomas. J Neurooncol 78: 281–293. doi:10.1007/s11060-005-9097-6. PubMed: 16554966. 16554966

[23] Li VW, Folkerth RD, Watanabe H, Yu C, Rupnick M et al. (1994) Microvessel count and cerebrospinal fluid basic fibroblast growth factor in children with brain tumours. Lancet 344: 82–86. doi:10.1016/S0140-6736(94)91280-7. PubMed: 7516992. 7516992

[24] Hunter T (2009) Tyrosine phosphorylation: thirty years and counting. Curr Opin Cell Biol 21: 140–146. doi:10.1016/j.ceb.2009.01.028. PubMed: 19269802. 19269802 PMC2670436

[25] Verhaak RGW, Hoadley KA, Purdom E, Wang V, Qi Y et al. (2010) Integrated genomic analysis identifies clinically relevant subtypes of glioblastoma characterized by abnormalities in PDGFRA, IDH1, EGFR, and NF1. Cancer Cell 17: 98–110. doi:10.1016/j.ccr.2009.12.020. PubMed: 20129251. 20129251 PMC2818769

[26] Eliceiri BP, Paul R, Schwartzberg PL, Hood JD, Leng J et al. (1999) Selective requirement for Src kinases during VEGF-induced angiogenesis and vascular permeability. Mol Cell 4: 915–924. doi:10.1016/S1097-2765(00)80221-X. PubMed: 10635317. 10635317

[27] Weis S, Shintani S, Weber A, Kirchmair R, Wood M et al. (2004) Src blockade stabilizes a Flk/cadherin complex, reducing edema and tissue injury following myocardial infarction. J Clin Invest 113: 885–894. doi:10.1172/JCI20702. PubMed: 15067321. 15067321 PMC362122

[28] Abu-Ghazaleh R, Kabir J, Jia H, Lobo M, Zachary I (2001) Src mediates stimulation by vascular endothelial growth factor of the phosphorylation of focal adhesion kinase at tyrosine 861, and migration and anti-apoptosis in endothelial cells. Biochem J 360: 255–264. doi:10.1042/0264-6021:3600255. PubMed: 11696015. 11696015 PMC1222225

[29] Imamoto A, Soriano P (1993) Disruption of the csk gene, encoding a negative regulator of Src family tyrosine kinases, leads to neural tube defects and embryonic lethality in mice. Cell 73: 1117–1124. doi:10.1016/0092-8674(93)90641-3. PubMed: 7685657. 7685657

[30] Pedraza LG, Stewart RA, Li D-M, Xu T (2004) Drosophila Src-family kinases function with Csk to regulate cell proliferation and apoptosis. Oncogene 23: 4754–4762. doi:10.1038/sj.onc.1207635. PubMed: 15107833. 15107833

[31] Potter MD, Barbero S, Cheresh DA (2005) Tyrosine phosphorylation of VE-cadherin prevents binding of p120- and beta-catenin and maintains the cellular mesenchymal state. J Biol Chem 280: 31906–31912. doi:10.1074/jbc.M505568200. PubMed: 16027153. 16027153

[32] Baumeister U, Funke R, Ebnet K, Vorschmitt H, Koch S et al. (2005) Association of Csk to VE-cadherin and inhibition of cell proliferation. EMBO J 24: 1686–1695. doi:10.1038/sj.emboj.7600647. PubMed: 15861137. 15861137 PMC1142580

[33] Plate KH, Breier G, Weich HA, Risau W (1992) Vascular endothelial growth factor is a potential tumour angiogenesis factor in human gliomas in vivo. Nature 359: 845–848. doi:10.1038/359845a0. PubMed: 1279432. 1279432

[34] Jain RK, di Tomaso E, Duda DG, Loeffler JS, Sorensen AG et al. (2007) Angiogenesis in brain tumours. Nat Rev Neurosci 8: 610–622. doi:10.1038/nrn2175. PubMed: 17643088. 17643088

[35] Haas TL, Madri JA (1999) Extracellular matrix-driven matrix metalloproteinase production in endothelial cells: implications for angiogenesis. Trends Cardiovasc Med 9: 70–77. doi:10.1016/S1050-1738(99)00014-6. PubMed: 10578520. 10578520

[36] Rao JS (2003) Molecular mechanisms of glioma invasiveness: the role of proteases. Nat Rev Cancer 3: 489–501. doi:10.1038/nrc1121. PubMed: 12835669. 12835669

[37] Brasch J, Harrison OJ, Ahlsen G, Carnally SM, Henderson RM et al. (2011) Structure and binding mechanism of vascular endothelial cadherin: a divergent classical cadherin. J Mol Biol 408: 57–73. doi:10.1016/j.jmb.2011.01.031. PubMed: 21269602. 21269602 PMC3084036

[38] Hormigo A, Gutin PH, Rafii S (2007) Tracking normalization of brain tumor vasculature by magnetic imaging and proangiogenic biomarkers. Cancer Cell 11: 6–8. doi:10.1016/j.ccr.2006.12.008. PubMed: 17222788. 17222788 PMC2952447

[39] Greenfield JP, Jin DK, Young LM, Christos PJ, Abrey L et al. (2009) Surrogate markers predict angiogenic potential and survival in patients with glioblastoma multiforme. Neurosurgery 64: 819–827; discussion: 10.1227/01.NEU.0000343742.06625.DB. PubMed: 19404145. 19404145

[40] Dunn GP, Rinne ML, Wykosky J, Genovese G, Quayle SN et al. (2012) Emerging insights into the molecular and cellular basis of glioblastoma. Genes Dev 26: 756–784. doi:10.1101/gad.187922.112. PubMed: 22508724. 22508724 PMC3337451

[41] Feng H, Hu B, Liu K-W, Li Y, Lu X et al. (2011) Activation of Rac1 by Src-dependent phosphorylation of Dock180(Y1811) mediates PDGFRα-stimulated glioma tumorigenesis in mice and humans. J Clin Invest 121: 4670–4684. doi:10.1172/JCI58559. PubMed: 22080864. 22080864 PMC3223070

[42] Chi AS, Sorensen AG, Jain RK, Batchelor TT (2009) Angiogenesis as a therapeutic target in malignant gliomas. Oncologist 14: 621–636. doi:10.1634/theoncologist.2008-0272. PubMed: 19487335. 19487335 PMC4790121

[43] Farias-Eisner G, Bank AM, Hwang BY, Appelboom G, Piazza MA et al. (2012) Glioblastoma biomarkers from bench to bedside: advances and challenges. Br J Neurosurg 26: 189–194. doi:10.3109/02688697.2011.629698. PubMed: 22176646. 22176646

[44] Tabatabai G, Stupp R, van den Bent MJ, Hegi ME, Tonn JC et al. (2010) Molecular diagnostics of gliomas: the clinical perspective. Acta Neuropathol 120: 585–592. doi:10.1007/s00401-010-0750-6. PubMed: 20862485. 20862485

[45] Charles NA, Holland EC, Gilbertson R, Glass R, Kettenmann H (2011) The brain tumor microenvironment. Glia 59: 1169–1180. doi:10.1002/glia.21136. PubMed: 21446047. 21446047

[46] Nagy JA, Feng D, Vasile E, Wong WH, Shih S-C, et al. (2006) Permeability properties of tumor surrogate blood vessels induced by VEGF-A. Lab Invest 86: 767–780 doi:10.1038/labinvest.3700436. 16732297

[47] Conjugated eicosapentaenoic acid inhibits vascular en... [ J Nutr. 2007] -——.NCBI (n.d.). Available: http://www.ncbi.nlm.nih.gov/pubmed/17311953. Accessed 24 June 2013 10.1093/jn/137.3.641 17311953

[48] Sawlani RN, Raizer J, Horowitz SW, Shin W, Grimm SA et al. (2010) Glioblastoma: a method for predicting response to antiangiogenic chemotherapy by using MR perfusion imaging--pilot study. Radiology 255: 622–628. doi:10.1148/radiol.10091341. PubMed: 20413772. 20413772 PMC2858811

[49] Jiang Z, Le Bas J-F, Grand S, Salon C, Pasteris C et al. (2011) Prognostic value of perfusion MR imaging in patients with oligodendroglioma: A survival study. J Neuroradiol 38: 53–61. doi:10.1016/j.neurad.2010.03.004. PubMed: 20554324. 20554324

[50] Sitohy B, Nagy JA, Jaminet S-CS, Dvorak HF (2011) Tumor-surrogate blood vessel subtypes exhibit differential susceptibility to anti-VEGF therapy. Cancer Res 71: 7021–7028. doi:10.1158/0008-5472.CAN-11-1693. PubMed: 21937680. 21937680 PMC3217088

[51] Petricoin EF, Belluco C, Araujo RP, Liotta LA (2006) The blood peptidome: a higher dimension of information content for cancer biomarker discovery. Nat Rev Cancer 6: 961–967. doi:10.1038/nrc2011. PubMed: 17093504. 17093504

[52] Ebos JML, Lee CR, Bogdanovic E, Alami J, Van Slyke P et al. (2008) Vascular endothelial growth factor-mediated decrease in plasma soluble vascular endothelial growth factor receptor-2 levels as a surrogate biomarker for tumor growth. Cancer Res 68: 521–529. doi:10.1158/0008-5472.CAN-07-3217. PubMed: 18199548. 18199548

