# Supplementary material for: Evidence for Post-Translational Processing of Vascular Endothelial (VE)-Cadherin in Brain Tumors: Towards a Candidate Biomarker
Source: PLoS One. 2013 Dec 16;8(12):e80056. doi: 10.1371/journal.pone.0080056 (PMC3864785; doi:10.1371/journal.pone.0080056)

**SUPPLEMENTARY INFORMATION**

**Supplementary Methods**

**Mammalian protein production**

Human glycosylated VE-cadherin extracellular domain was produced as described in (Brasch J et al, 2011). The exact VE-cadherin concentration was determined using mass spectrometry analysis (430μg/mL).

**ELISA for soluble VE-cadherin detection in human serum**

The assay is a sandwich enzyme immunoassay using a monoclonal antibody and an enzyme-linked polyclonal antibody specific for sVE-cadherin. The capture monoclonal antibody has been pre-coated onto a 96-wells microplate overnight (ON) at 4°C. After a 1h period of incubation at room temperature (RT) with 100 μL of block buffer followed by washing (3 times), 100 μL of calibration, validation and human serum samples were added. After a 2h period of incubation, unbounded substances were removed by a second washing. 100 μL of the second antibody (Anti-Cad 3 IgG) were added to the wells and after a third incubation of 1 hour followed by washing. 100 μL of a polyclonal goat anti-rabbit immunoglobulins biotinylated were added. After 1h of incubation followed by washing, 100 μL of streptavidin-alkaline phosphatase were dispensed for 30 minutes. After washing, 200 μL of the substrate (pNPP) were added. During incubation, color developed in proportion to the amount of sVE-cadherin bounded in the initial step. The color development was followed up to 30 minutes and the enzymatic reaction was stopped by the addition of NaOH 3N. The absorbance was red at 405 nm using an ELISA reader (Benchmark microplate reader, Bio-Rad Laboratories, Hercules, California) and compatible software (Microplate Manager 5.2, Bio-Rad). All the analyses were performed in duplicate. The calibration range was from 2.5 to 40.0 ng/mL. Analytical data treatment: The calculations were performed independently. Raw data (OD) measured by the plate reader were plotted against nominal standard concentrations to construct the standard calibration curves. Concentration values of unknown samples were interpolated from these curves using an unweighted linear regression of the data. A typical standard curve is presented in Supplementary Figure 1. Throughout the study, the CVs of standards were from 1.26% to 9.54%. No cross-reactivity between soluble VE-cadherin and other adhesion molecules has been found using this assay. Experiments were performed in triplicate. The patient’s sera (5 μl) were diluted 1:100 dilution in 0.5% Triton X100-containing PBS. Individual serum concentrations for VE-Cadherin are reported in ng/mL.

**SUPPLEMENTARY TABLES**

| **Assay No.** | **Assay**  **date** | **Nominal concentrations of standard (ng/mL)** | | | | | | |
| --- | --- | --- | --- | --- | --- | --- | --- | --- |
|  |  | **ST1**  **1.25** | **ST2**  **2.50** | **ST3**  **5.00** | **ST4**  **7.50** | **ST5**  **10.00** | **ST6**  **15.00** | **ST7**  **20.00** |
| 1 | 12-Mars-12 | 1.23 | 2.46 | 6.59 | 7.98 | 10.47 | 15.14 | 19.12 |
| 2 | 13-Mars-12 | 1.07 | 2.43 | 6.82 | 9.65 | 9.85 | 15.23 | 18.70 |
| 3 | 14-Mars-12 | 0.85 | 2.66 | 5.52 | 6.96 | 9.54 | 14.82 | 20.65 |
| 4 | 15-Mars-12 | 0.84 | 1.99 | 4.93 | 8.95 | 10.86 | 15.08 | 19.10 |
| 5 | 15-Mars-12 | 1.18 | 2.63 | 5.78 | 8.62 | 10.22 | 15.14 | 19.20 |
| 6 | 19-Mars-12 | 1.02 | 2.41 | 5.03 | 9.40 | 10.60 | 15.07 | 18.91 |
| Mean | | 1.03 | 2.43 | 5.78 | 8.59 | 10.26 | 15.08 | 19.28 |
| S.D. | | 0.15 | 0.22 | 0.72 | 0.9 | 0.45 | 0.13 | 0.63 |
| %CV | | 14.70 | 9.00 | 12.40 | 10.50 | 4.37 | 0.84 | 3.29 |
| n | | 6 | 6 | 6 | 6 | 6 | 6 | 6 |
| min | | 0.84 | 1.99 | 4.93 | 6.96 | 9.54 | 14.82 | 18.70 |
| max | | 1.23 | 2.66 | 6.82 | 9.65 | 10.86 | 15.23 | 20.65 |
| %Diff | | 17.6 | 2.8 | -15.6 | -14.5 | -2.6 | -0.5 | 3.6 |

**Supplementary Table S1 :** Between assay – sVE-cadherin back-calculated concentrations (ng/mL) of standards (ST). S.D: standard deviation; %CV: percent of coefficient of variation; %Diff: percent of difference between the calculated and the nominal concentrations.

| **Assay No.** | **Assay date** | **Concentrations (ng/mL) of validation samples** | | | | |
| --- | --- | --- | --- | --- | --- | --- |
|  |  | **Low**  **VE-cad** | **High**  **VE-cad** | **EFS**  **1178** | **EFS**  **1207** | **EFS**  **pool** |
| 1 | 12-Mars-12 | 267.9 | 1635.1 | 351.2 | 454.7 | 534.9 |
| 2 | 13-Mars-12 | 265.1 | 1520.6 | 428.0 | 444.3 | 462.2 |
| 3 | 14-Mars-12 | 267.6 | 14.56.2 | 485.4 | 441.1 | 596.2 |
| 4 | 15-Mars-12 | 237.1 | 1405.5 | 383.8 | 327.0 | 508.8 |
| 5 | 15-Mars-12 | 273.0 | 1480.3 | 379.6 | 500.6 | 608.3 |
| 6 | 19-Mars-12 | 262.9 | 1417.8 | 325.2 | 489.3 | 405.1 |
| Mean | | 262.3 | 1485.9 | 392.2 | 442.8 | 519.3 |
| S.D. | | 11.7 | 76.9 | 52.2 | 56.4 | 71.3 |
| %CV | | 4.5 | 5.2 | 13.3 | 12.7 | 13.7 |
| n | | 6 | 6 | 6 | 6 | 6 |
| %Diff | | -4.9 | 0.9 | 12.8 | 11.4 | 5.6 |

**Supplementary Table S2**: Between assay – sVE-cadherin back-calculated concentrations (ng/mL) of validation samples. S.D: standard deviation; %CV: percent of coefficient of variation; %Diff: percent of difference between the calculated and the nominal concentrations.

| **Serum of glioma patients (ng/mL)** | **Assay date** | | |  |  |  |  |
| --- | --- | --- | --- | --- | --- | --- | --- |
|  | 29-Mars-12 | 30-Mars-12 | 12-April-12 | Mean | S.D. | %CV | n |
| **1** | 870 | 1173 | 1035 | 1026 | 123.8 | 12.07 | 3 |
| **2** | 1302 | 1377 | 1220 | 1300 | 64.11 | 4.93 | 3 |
| **3** | 976 | 1236 | 986 | 1066 | 120.27 | 11.25 | 3 |
| **4** | 851 | 914 | 655 | 807 | 110.28 | 13.66 | 3 |
| **5** | 881 | 972 | 850 | 901 | 51.77 | 5.74 | 3 |
| **6** | 979 | 939 | 1111 | 1009 | 73.49 | 7.28 | 3 |
| **7** | 1090 | 1296 | 1240 | 1209 | 86.97 | 7.19 | 3 |
| **8** | 1382 | 1338 | 1240 | 1320 | 59.35 | 4.49 | 3 |
| **9** | 1371 | 1401 | 1364 | 1379 | 16.05 | 1.16 | 3 |
| **10** | 759 | 821 | 730 | 770 | 37.95 | 4.92 | 3 |
| **11** | 419 | 524 | 580 | 508 | 66.73 | 13.13 | 3 |
| **12** | 987 | 966 | 996 | 983 | 12.57 | 1.28 | 3 |
| **13** | 865 | 716 | 893 | 825 | 77.68 | 9.41 | 3 |
| **14** | 810 | 710 | 685 | 735 | 54.00 | 7.34 | 3 |
| **15** | 810 | 776 | 1007 | 864 | 100.83 | 11.67 | 3 |
| **16** | 706 | 581 | 697 | 661 | 56.99 | 8.61 | 3 |
| **17** | 635 | 629 | 565 | 610 | 31.67 | 5.19 | 3 |
| **18** | 414 | 419 | 431 | 455 | 54.01 | 11.88 | 3 |
| **19** | 324 | 392 | 315 | 343 | 34.37 | 10.02 | 3 |
| **20** | 628 | 758 | 839 | 741 | 86.91 | 11.72 | 3 |

**Supplementary Table S3**: Large batch size evaluation (inter-day assay) of human serum samples (ng/mL).20 serum of glioma patients were analysed in three different days. S.D: standard deviation; %CV: percent of coefficient of variation.

| **Assay date** | **Storage** | **Concentration (ng/mL)** |
| --- | --- | --- |
|  |  | **Human serum** |
| 12-April-12 | T0 | 655.8 |
|  |  | 629.4 |
|  |  | 682.2 |
|  | Mean | 655.8 |
|  | S.D. | 21.55 |
|  | %CV | 3.28 |
| 12-April-12 | 2 hours at RT | 529.4 |
|  |  | 526.9 |
|  |  | 532.0 |
|  | Mean | 529.4 |
|  | S.D. | 2.00 |
|  | %CV | 0.39 |
|  | %Loss | 19.27 |
| 12-April-12 | 24 hours at RT | 504.1 |
|  |  | 506.6 |
|  |  | 501.5 |
|  | Mean | 504.1 |
|  | S.D. | 2.8 |
|  | %CV | 0.41 |
|  | %Loss | 24.28 |
|  | | |

**Supplementary Table S4**: Evaluation of sVE-cadherin stability in human human serum at room temperature (RT) up to 24 hours. Serum samples (ng/mL). S.D: standard deviation; %CV: percent of coefficient of variation; %Loss calculated in relation to the T0 value.

**LEGENDS TO SUPPLEMENTARY TABLES AND FIGURES**

**Supplementary Figure S1: Characterization of the human recombinant glycosylated VE-cadherin ectodomain.** (A) Production of calibration standard by EBNA eukaryotic cells. A cDNA fragment containing the sequence encoding the EC1-5 fragment of human VE-cadherin was produced by PCR. After digestion, the fragment was inserted in the pCEP4 plasmid. The glycosylated sVE was produced in EBNA cells. Analysis of the conditioned medium by Western blot with BV9 antibody is illustrated. (B) PGNase treatment of recombinant VE1-5g shows that the protein is glycosylated. Deglycosylation assay: 50 and 200 ng of the recombinant protein were treated or not (- ; +) as indicated. Control samples were treated with the same buffers in the absence of enzyme. 20 μl of the mixture was analyzed by SDS-PAGE and WB with VE-cadherin antibody (BV9). (C) Glycosylated VE-cadherin. Analysis of the recombinant protein by SDS-PAGE and Coomassie staining. (D) ELISA for quantitative detection of sVE-cadherin in human serum. (Step 1) Antibody (BV9) is immobilized on microwell plates; (step 2) VE1-5g is captured by BV9; (Step 3) rabbit polyclonal anti-Cad3 antibody visualized by (step 4) biotin-conjugate goat anti-rabbit IgG and (step 5) streptavidin-alkaline phosphatase. The unbound material is washed each step. (step 6) an antigen-specific antibody conjugated to enzyme (i.e., developing reagent) is added. Recombinant human glycosylated VE-Cadherin produced by transfection of mammalian cells and quantified by mass spectrometry was used to prepare VE-cadherin calibration standard curve. A representative standard curve from human VE1-5g fragment was obtained by serial dilution in assay buffer. Each point on the graph represents the mean of the three parallel titrations and dilution linearity has been already reported in the patent.

**Supplementary table S1: Between assay - sVE-cadherin back-calculated concentrations (ng/mL) of standards.**

The detection of a soluble form of glycosylated VE-cadherin in the human serum was highly improved using a detergent-containing buffer. The recombinant human VE-cadherin (VE1-5g) at 430 µg/ml was stored at -80°C. Additional VE-cadherin calibration standards are made by dilution of the standard to 20.0 µg/mL using 41 µL of the assay buffer each day as needed. Seven points of calibration standards (ST) were obtained as shown in the table. The addition of detergent in the assay buffer allowed to perform a linear calibration curve. The acceptance criteria for the 7 calibration standards, for precision and accuracy expressed as %CV and %Diff respectively, was set at 15%

**Supplementary table S2: Between assay - sVE-cadherin back-calculated concentrations (ng/mL) of validation samples.**

Validation samples (called Low VE-cad, High VE-cad, C3, EFS 1178, EFS 1207, EFS pool) were collected in 2012. These samples are kept at -80°C and diluted extemporarily. Low VE-cad and High VE-cad samples were selected from glioma patients having low or high sVE-cadherin concentration. They were defined as 250 and 1500 ng/mL, respectively. In addition, we selected healthy donors (EFS 1178, EFS 1208, EFS pool) as 450, 500, 550 ng/mL, respectively. sVE-cadherin concentrations determined in human serum samples from the these 3 validation groups are reported to give an estimate of the variability of normal values. For between-assay performance characterization, the five validation samples were assayed over 5 days (6 runs). In each analysis, there was a blank and all the concentrations were back calculated with the first set of standards. The acceptance criteria for the 5 validation samples, the %CV and %Diff were set and obtained at 20%.

**Supplementary table S3: Large batch size evaluation (Inter-day assay) of human serum samples (ng/mL).** 20 serums of glioma patients were analyzed in three different days. During the large batch size evaluation of 20 glioma patient serums, the %CV was no more than 13.66.

**Supplementary table S4: Evaluation of sVE-cadherin stability in human serum at room temperature up to 24 hours.**

The stability of sVE-cadherin in human serum (EFS pool) was tested after 2 and 24 hours at RT and after thawing 5 minutes (T0) at RT before the assay quantification. The %Diff was calculated using the T0 value as reference. STable 4 ilustrate the results of the stability assay for sVE-cadherin in human serum and precision was from 0.39 to 3.28%. All aliquots of human serum samples were stored at ‑80°C. The stability of sVE-cadherin was tested upon the following conditions: (1) thawing and storage at RT for 2h before sVE-cadherin quantification; (2) thawing and storage at RT for 24h before sVE-cadherin assay. The results illustrated showed a partial loss of sVE-cadherin immunoreactivity in conditions 1 and 2 (19.27 and 24.28%) when compared to T0. This analytical method corresponds to a prototype assay, which suggests carefull steps during performance. Therefore, aliquots of serum samples and glycosylated VE1-5g calibration standards are stored at -80°C and thaw each time before use.

**SUPPLEMENTARY FIGURES**

**Supplementary Figure S1**: Production of human recombinant glycosylated sVE-cadherin in EBNA cells and development of standard calibration curve for ELISA quantification of sVE-cadherin in human serum


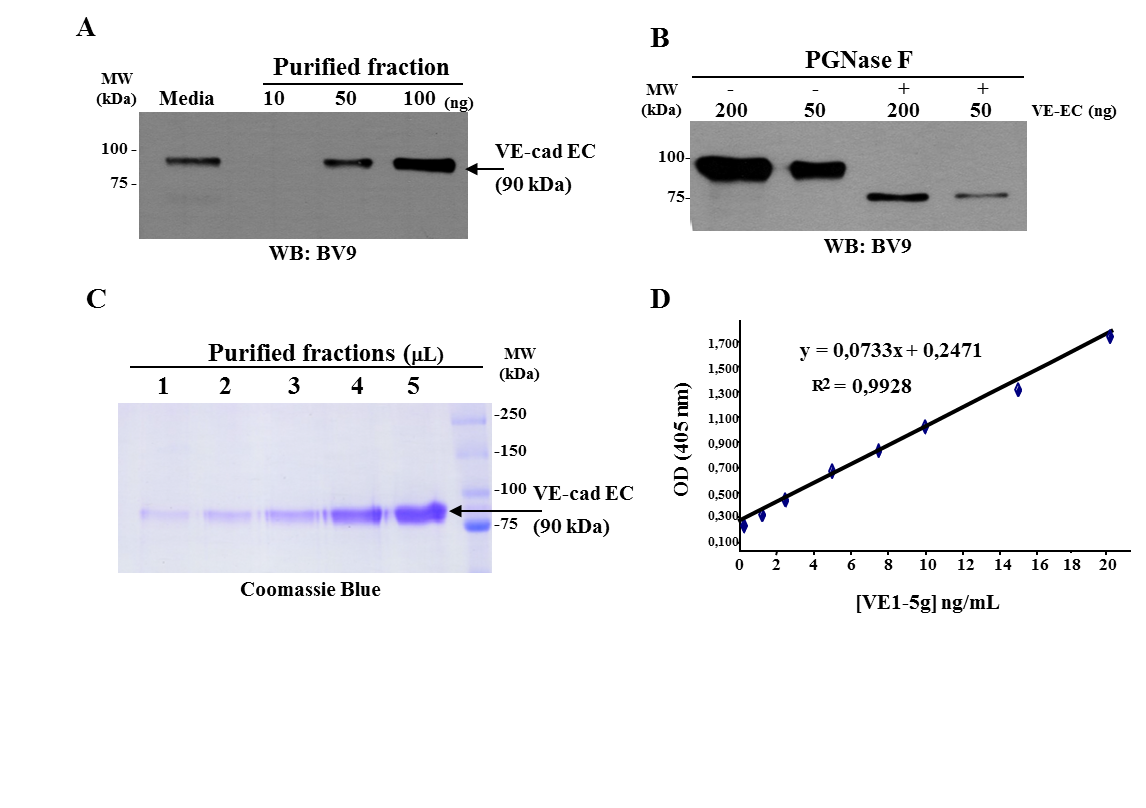

Supplement: Methods S1 — (DOCX) [file pone.0080056.s001.docx]
